# Supplementary material for: Glucocorticoid-Regulated Kinase CAMKIγ in the Central Amygdala Controls Anxiety-like Behavior in Mice
Source: Int J Mol Sci. 2022 Oct 14;23(20):12328. doi: 10.3390/ijms232012328 (PMC9604347; doi:10.3390/ijms232012328)
Supplement: Supplementary file 1 [file ijms-23-12328-s001.zip › ijms-1921843-supplementary.pdf]

**Supplementary Figures**

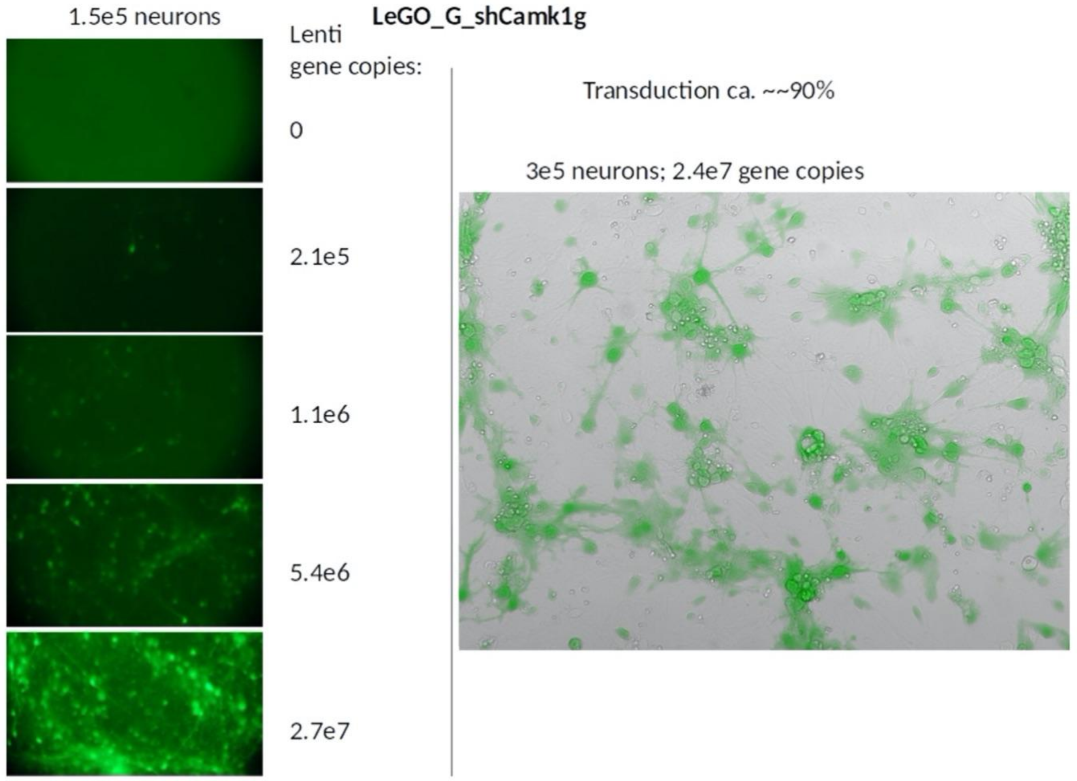


**Supplementary Figure S1.** Transfection efficiency based on the number of lentiviral particles used for primary neuronal culture transduction.

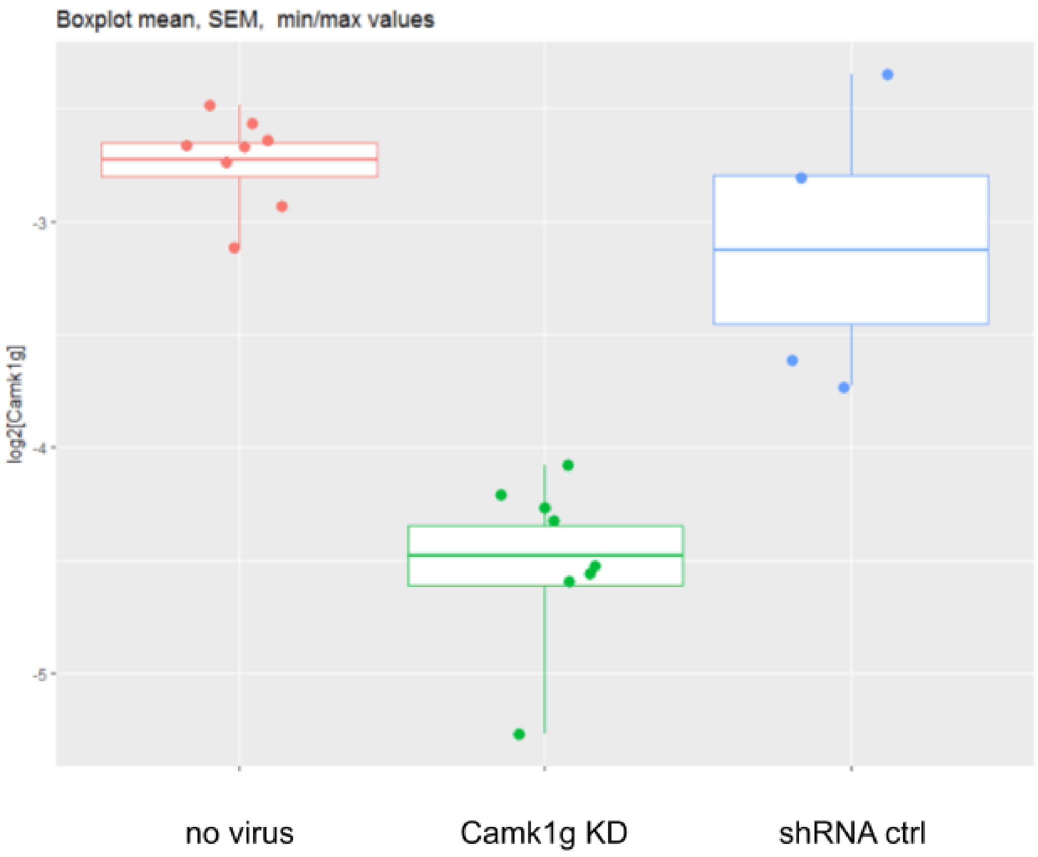


**Supplementary Figure S2**. Camk1g KD in primary neuronal cultures as validated by using qPCR. One-way ANOVA p-value 2.76e-07 ***, pairwise t-test p-values: Camk1g KD vs no virus 2.6e-07 ***, Camk1g KD vs shRNA ctrl 6.3e-05 ***, no virus vs shRNA ctrl 0.12.

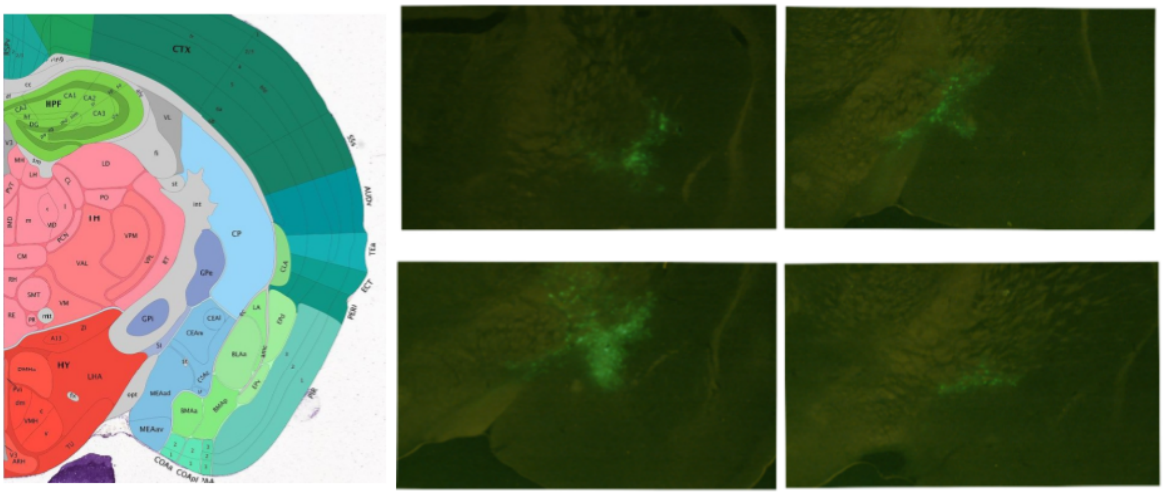


**Supplementary Figure S3.** The precision of injection of lentiviral shRNA for *Camk1g* into the CeA. Left panel - image from the Allen mouse brain atlas showing the location of the slices where the images were taken. Right panel - images from four representative animals are shown. Green - GFP signal from the transgene.

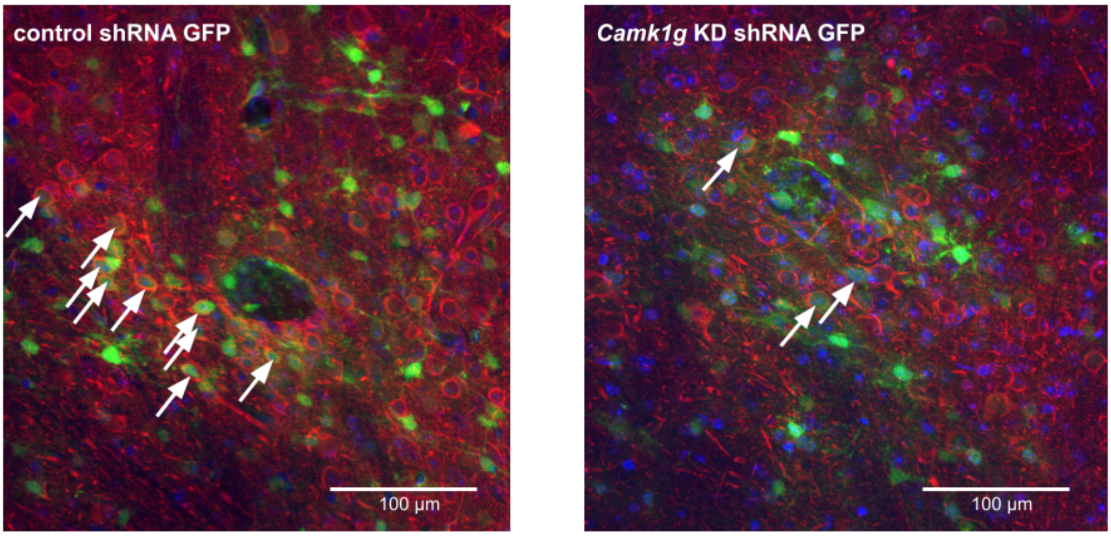


**Supplementary Figure S4.** Representative immunofluorescence images of Camk1g KD effect in the CeA. Red - anti-Camk1g staining, green - GFP signal, blue - DAPI signal. Arrows point to cells having both GFP and anti-Camk1g signals. The number of cells positive for anti-Camk1g and GFP is lower in Camk1g KD animals and is generally associated with weak GFP fluorescence.
